# Game Theory of Social Distancing in Response to an Epidemic

**DOI:** 10.1371/journal.pcbi.1000793

**Published:** 2010-05-27

**Authors:** Timothy C. Reluga

**Affiliations:** Department of Mathematics, Pennsylvania State University, State College, Pennsylvania, United States of America; University of Washington, United States of America

## Abstract

Social distancing practices are changes in behavior that prevent disease transmission by reducing contact rates between susceptible individuals and infected individuals who may transmit the disease. Social distancing practices can reduce the severity of an epidemic, but the benefits of social distancing depend on the extent to which it is used by individuals. Individuals are sometimes reluctant to pay the costs inherent in social distancing, and this can limit its effectiveness as a control measure. This paper formulates a differential-game to identify how individuals would best use social distancing and related self-protective behaviors during an epidemic. The epidemic is described by a simple, well-mixed ordinary differential equation model. We use the differential game to study potential value of social distancing as a mitigation measure by calculating the equilibrium behaviors under a variety of cost-functions. Numerical methods are used to calculate the total costs of an epidemic under equilibrium behaviors as a function of the time to mass vaccination, following epidemic identification. The key parameters in the analysis are the basic reproduction number and the baseline efficiency of social distancing. The results show that social distancing is most beneficial to individuals for basic reproduction numbers around 2. In the absence of vaccination or other intervention measures, optimal social distancing never recovers more than 30% of the cost of infection. We also show how the window of opportunity for vaccine development lengthens as the efficiency of social distancing and detection improve.

## Author Summary

One of the easiest ways for people to lower their risk of infection during an epidemic is for them to reduce their rate of contact with infectious individuals. However, the value of such actions depends on how the epidemic progresses. Few analyses of behavior change to date have accounted for how changes in behavior change the epidemic wave. In this paper, I calculate the tradeoff between daily social distancing behavior and reductions in infection risk now and in the future. The subsequent analysis shows that, for the parameters and functional forms studied, social distancing is most useful for moderately transmissible diseases. Social distancing is particularly useful when it is inexpensive and can delay the epidemic until a vaccine becomes widely available. However, the benefits of social distancing are small for highly transmissible diseases when no vaccine is available.

## Introduction

Epidemics of infectious diseases are a continuing threat to the health of human communities, and one brought to prominence in the public mind by the 2009 pandemic of H1N1 influenza [1]. One of the key questions of public health epidemiology is how individual and community actions can help mitigate and manage the costs of an epidemic. The basic problem I wish to address here is how rational social-distancing practices used by individuals during an epidemic will vary depending on the efficiency of the responses, and how these responses change the epidemic as a whole.

Social distancing is an aspect of human behavior particularly important to epidemiology because of its universality; everybody can reduce their contact rates with other people by changing their behaviors, and reduced human contact reduces the transmission of many diseases. Theoretical work on social distancing has been stimulated by studies of agent-based influenza simulations indicating that small changes in behavior can have large effects on transmission patterns during an epidemic [2]. Further research on agent-based models has argued that social distancing can arrest epidemics if started quickly and maintained for a relatively long period [3]. Compartmental epidemic models have also been used to study social distancing by including states that represent individuals employing specific behaviors. For instance, Hyman and Li [4] formulate and begin the analysis of flu disease transmission in SIR models where some individuals decrease their activity levels following infection. Reluga and Medlock [5] uses this approach to show that while social distancing can resemble immunization, it can generate hysteresis phenomena much more readily than immunization.

Rather than treating behaviors as states, some models treat behaviors as parameters determined by simple functions of the available information. Reluga et al. [6] studies dynamics where contact rates can depend on the perceived disease incidence. Buonomo et al. [7] investigates the impact of information dynamics on the stability of stationary solutions in epidemic models. Chen [8] considers a similar system but allows individuals to learn from a random sample of neighbors. Funk et al. [9] considers the information dynamics associated with social distancing in a network setting by prescribing a reduction in contacts based on proximity to infection. Related work by Epstein et al.[10] explicitly considers the spatial and information dynamics associated in response to an ongoing epidemic.

Building on the ground-breaking work of Fine and Clarkson [11], there has been substantial recent interest in the application of game theory to epidemiology [12]–[17]. The games studied so far have primarily considered steady-state problems, and have not allowed for dynamic strategies. One notable exception to this is the work of Francis [18], which determines the time-dependent game-theoretical solution of a vaccination problem over the course of an epidemic. In another, van Boven et al. [19] studies the optimal use of anti-viral treatment by individuals when they take into account the direct and indirect costs of treatment.

To study the best usage of social distancing, we apply differential-game theory at a population-scale. Differential games are games where strategies have a continuous time-dependence; at each point in time, a player can choose a different action. For instance, a pursuit-game between a target and a pursuer is a two-player differential game where each player's strategies consist of choosing how to move at each successive time until the target is caught by the pursuer or escapes. Geometrically, one might think of differential games as games where strategies are represented by curves instead of points. Two-player differential-game theory was systematically developed by Isaacs [20] as an extension of optimal control theory [21]–[23]. Here, we employ an extension of differential game theory to population games of the form described by Reluga and Galvani [24]. The analysis in this paper will be limited to the simplest case of the Kermack–McKendrick SIR model with strong mixing [25].

In the Model section, we formulate an epidemiological-economics model for an epidemic, accounting for the individual and community costs of both social distancing practices and infection. We then use differential game theory and numerical methods to identify the equilibrium strategies over the course of an epidemic. Numerical methods are used to investigate the finite-time problem where vaccines become available after a fixed interval from the start of the epidemic and the infinite-horizon problem without vaccination. Fundamental results on the value and timing of social distancing are obtained.

## Model

In this article, social distancing refers to the adoption of behaviors by individuals in a community that reduce those individuals' risk of becoming infected by limiting their contact with other individuals or reducing the transmission risk during each contact. Typically, social distancing incurs some costs in terms of liberty, social capital, time, convenience, and money, so that people are only likely to adopt these measures when there is a specific incentive to do so. In addition to the personal consequences, the aggregate effects of social distancing form an economic externality, reducing the overall transmission of disease. This externality needs to be accounted for in the determination individuals optimal strategies, but, by definition, depends on the choice of strategy.

To resolve this interdependence, we formulate our analysis as a population game where the payoff to each individual is determined by the individual's behavioral strategy and the average behavioral strategy used by the population as a whole. The model is related to that previously studied by Chen [26]. We will use 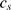 to represent one specific individual's strategy of daily investment in social distancing. The population strategy 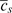 is the aggregate daily investment in social distancing by the population. The overbar notation is used to indicate that the aggregate investment 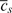 should be thought of as an average investment aggregated over all individuals in the population. In the limit of infinitely large populations, 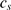 and 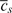 can be thought of as independent because changes in one person's behavior will have little affect on the average behavior. Similarly, the epidemic's dynamics depend on the population strategy 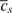 but are independent of any one individual's behavior 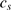.

The effectiveness of social distancing is represented by a function 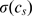, which is the relative risk of infection given a daily investment 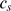 in social distancing practices. If there is no investment, the relative risk 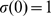. As the daily investment 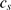 increases, the relative risk 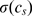 decreases, but is bounded below by 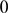. We expect diminishing returns with increasing investment, so we will also make the convenient assumption that 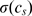 is convex.

Consider a Susceptible-Infected-Recovered (SIR) epidemic model with susceptible (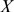), infected and infectious (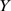), and removed (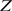) states. Suppose an epidemic starts with 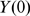 cases in a community of 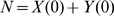 total individuals (taking 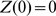) and proceeds until time 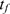, at which point all the individuals in the susceptible state are vaccinated. This epidemic is fast relative to demographic processes and we do not distinguish among the possible states of individuals leaving the infectious state, so the population size 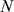 can be treated as constant. Between time 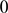 and time 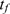, the dynamics are described by
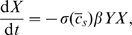
(1a)

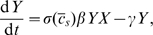
(1b)

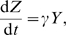
(1c)where 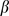 is the transmission rate and 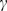 is the removal rate. This SIR model assumes the population is homogeneous, strongly mixed, and that the duration of infections is exponentially distributed. At the start of the epidemic when there are few cases of infection (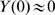), the basic reproduction number 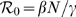.

The total cost of the epidemic to the community, 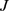, is the sum of the direct costs plus the indirect costs of any economic repercussions from the epidemic. To keep our analysis tightly focused, we will only consider direct costs of the epidemic, including the daily costs from infection, daily investments in social distancing, and the costs of vaccination. Mathematically,
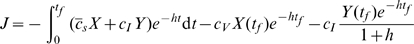
(2)where 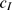 is the daily cost of each infection, 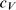 is the cost of vaccination per person, and 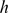 is the discount rate. Note that while the cost of infection 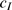 is a constant, the investment in social distancing 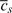 is a function of time. The last term in Eq. (2) is called a salvage term and represents the cumulative costs associated with individuals who are sick at the time the vaccine is made available (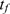). The assumption that the entire remaining susceptible population is vaccinated at time 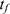 and that vaccination takes effect instantly is, of course, unrealistic, but does provide an approximation to the delayed release of a vaccine.

To simplify our studies, we will work with the dimensionless version of the equations by taking:
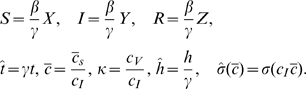
(3)Under this choice of units, time will be measured in terms of disease generations, social distancing costs will be measured relative to the daily cost of infection, and population sizes will be measured relative to the critical population size necessary to sustain an epidemic.

Epidemics usually start with one or a few index cases, so we focus on scenarios where 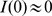. The dynamics can be described in terms the shape of 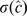, the discount rate 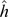, and a single initial-condition parameter
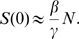
(4)From this, it follows that 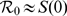. Since epidemics are often much faster than human demographic processes governing the discount rate [27], we will also take 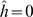 in all calculations. Henceforth, we will drop the hat-notation and work with the dimensionless parameters. The dimensionless equations are
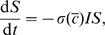
(5a)

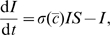
(5b)

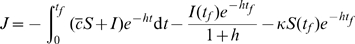
(5c)with the constraint that 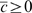. Note that we drop the function notation when necessary to simplify the presentation.

For our further analysis, we will assume
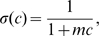
(6)with the maximum efficiency of social distancing 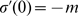. Eq. (6) is nicely behaved for numerical solutions because of its relatively fat tail.

### The Social Distancing Game

We now formulate a differential game for individuals choosing their best social distancing practices relative to the aggregate behavior of the population as a whole. The following game-theoretic analysis combines the ideas of Isaacs [20] and Reluga and Galvani [24]. The premise of the game is that at each point in the epidemic, people can choose to pay a cost associated with social distancing in exchange for a reduction in their risk of infection. The costs of an epidemic to the individual depend on the course of the epidemic and the individual's strategy of social distancing. The probabilities 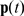 that an individual is in the susceptible, infected, or removed state at time 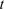 evolve according to the Markov process
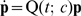
(7)where 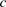 is the individual's daily investment as a function of the epidemic's state-variables and the transition-rate matrix
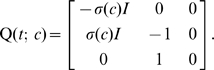
(8)Note that both 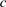 and 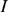 change over time. Along the lines discussed above, 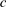 and 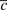 represent different quantities in our analysis; 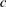 represents one individual's investment strategy and the population strategy 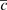 represents an aggregated average of all individual investments. We also note that there are several different ways 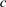 and 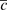 can be parameterized. They may be parameterized in terms of time, as 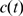 and 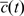, or in implicit feedback form 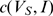 and 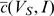, or in explicit feedback form 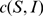 and 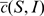. The form used will be clear from the context.

Since the events in the individual's life are stochastic, we can not predict the exact time spent in any one state or the precise payoff received at the end of the game. Instead, we calculate expected present values of each state at each time, conditional on the investment in social distancing. The expected present value is average value one expects after accounting for the probabilities of all future events, and discounting future costs relative to immediate costs. The expected present values 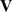 of each state evolve according to the adjoint equations
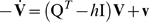
(9)where 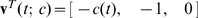. The components 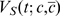, 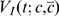, and 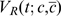 represent the expected present values of being in the susceptible, infected, or removed state at time 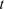 when using strategy 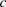 in a population using strategy 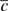. The expected present values depend on the population strategy 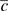 through the infection prevalence 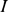.

The adjoint equations governing the values of each state are derived from Markov decision process theory. They are
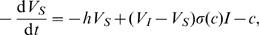
(10a)

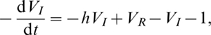
(10b)

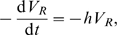
(10c)with the constraints that 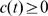 for all time 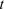. Solution of (10)b and (10)c gives
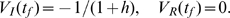
(11)If it is impossible to make a vaccine, the equations must be solved over an infinite horizon. Over an infinite horizon, 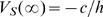, assuming 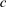 becomes constant. In the case of no discounting (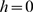), we still have 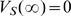 provided 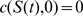 for sufficiently large 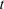. In the case where a perfect vaccine is universally available at terminal time 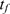, the value of the susceptible and removed states differs by the cost of vaccine 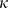 for 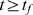. To avoid complications with the choice of whether-or-not to vaccinate, we take 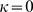 so 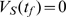. This is reasonable in scenarios where the cost of the vaccine is covered by the government.

The dynamics are independent of 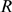, so we need not consider removed individuals further. Taking 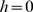 and 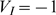, we need only study the reduced system
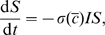
(12a)

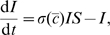
(12b)

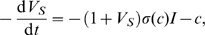
(12c)with boundary conditions
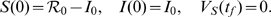
(12d)The other conditions must be calculated from the solution of the boundary-value problem and provide useful information. 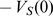 will be the expected total cost of the epidemic to the individual. The final size of the epidemic is given by 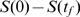.

### Game Analysis

Solving a game refers to the problem of finding the best strategy to play, given that all the other players are also trying to find a best strategy for themselves. In some games, there is a single strategy that minimizes a player's costs no matter what their opponents do, so that strategy can very reasonably be referred to as a solution. In many games, no such strategy exists. Rather, the best strategy depends on the actions of the other players. Any strategy played by one player is potentially vulnerable to a lack of knowledge of the strategies of the other players. In such games, it is most useful to look for strategies that are equilibria, in the sense that every player's strategy is better than the alternatives, given knowledge of their opponent's strategies. A Nash equilibrium solution to a population game like that described by System (12) is a strategy that is a best response, even when everybody else is using the same strategy. *i.e.* given 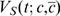, 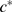 is a Nash equilibrium if for every alternative strategy 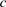, 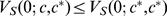. A Nash equilibrium strategy is a subgame perfect equilibrium if it is also a Nash equilibrium at every state the system may pass through. I will not address the problem of ruling out finite-time blowup of the Hamilton–Jacobi equation and establishing existence and uniqueness of subgame perfect equilibria. But numerical and analytical analyses strongly support the conjecture that the stategies calculated here are the unique global subgame perfect equilibria to the social distancing game.

**Figure 1:**
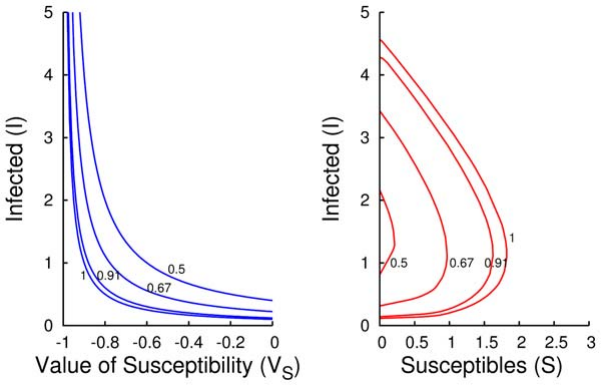
Contour plots of relative risk surface for equilibrium strategies. The relative risk is presented in feedback form with implicit coordinates 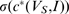 (left) and transformed to explicit coordinates 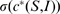 (right) for the infinite-horizon problem with maximum efficiency 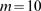. The greater the value of the susceptible state (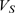), the greater the instantaneous social distancing. We find that increasing the number of susceptible individuals always decreases the investment in social distancing, and the greatest investments in social distancing occur when the smallest part of the population is susceptible. Note that in the dimensionless model, the value of the infection state 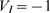.

The equilibria of System (12) can be calculated using the general methods of Isaacs [20]. The core idea is to implement a greedy-algorithm; at every step in the game, find the investment that maximizes the rate of increase in the individual's expect value 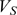. We represent strategies as functions in implicit feedback form. 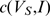 is the amount an individual invests per transmission generation when the system is at state 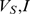. If 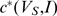 is a subgame perfect equilibrium, then it satisfies the maximum principle
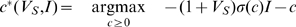
(13)when 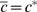 everywhere. So long as 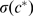 behaves well, in the sense that it is differentiable, decreasing, and strictly convex, then 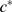 is uniquely defined by the relations
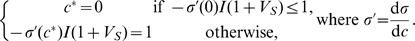
(14)
Figure 1 shows the interface in 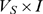 phase space separating the region where the equilibrium strategy will include no investment in social distancing (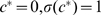) from the region where the equilibrium strategy requires investment in social distancing (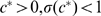).

Two cases are immediately interesting. The first is the infinite-horizon problem – what is the equilibrium behavior when there is never a vaccine and the epidemic continues on until its natural end? The second is the finite-horizon problem – if a vaccine is introduced at time 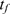 generations after the start of the epidemic, what is the optimal behavior while waiting for the vaccine? In both of these cases, it is assumed that all players know if and when the vaccine will be available.

The infinite-horizon and finite-horizon problems are distinguished by their boundary conditions. In the finite-horizon case, we assume all susceptible individuals are vaccinated at final time 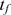, so 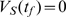, 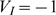, 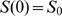, 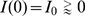 while 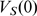 and 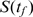 are unknown. In the limit of the infinite-horizon case (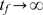), we solve the two-point boundary value problem with terminal conditions 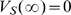, 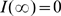, and initial conditions 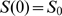, 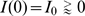 while 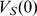 and 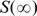 are unknown. But these conditions are insufficient to specify the infinite-horizon problem. The plane 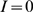 is a set of stationary solutions to Eq. (12), so we need a second order term to uniquely specify the terminal condition when we are perturbed slightly away from this plane. Using Eq. (12), we can show solutions solve the second-order terminal boundary condition
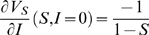
(15)for 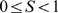 as 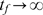.

Most of the equilibria we calculate are obtained numerically. Some exceptions are the special cases where 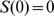, 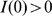. Under these conditions, solutions can be obtained in closed-form. First, 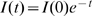. While 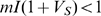, 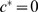 and
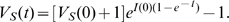
(16)When matched to the terminal boundary condition, we find that if we write 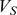 in feedback form as a function of 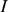 rather than 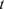,
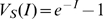
(17)is a solution so long as 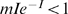 for all 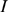. Inspecting the inequality condition, we find that this holds as long as 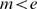.

## Results

A problem with solving Eq. (12) under Eq. (14) is that it requires 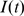 to be known from past time and 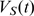 to be known from future time. This is a common feature of boundary-value problems, and is resolved by considering all terminal conditions 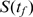. Using standard numerical techniques, identifying an equilibrium in the described boundary-value problem reduces to scalar root finding for 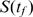 to match the given 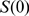. The special form of the population game allows the solution manifold to be calculated directly by integrating backwards in time, rather than requiring iterative approaches like those used for optimal-control problems [23]. Code for these calculations is available from the author on request.

Before presenting the results, it is helpful to develop some intuition for the importance of the maximum efficiency 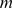 of investments in social distancing. Given 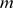 for an arbitrary relative risk function 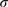, then in the best-case scenarios, where diminishments on returns are weakest, one would have to invest atleast 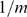 of the cost of infection per disease generation to totally isolate themselves. The units here are derived from dimensional analysis. This could be invested for no more than 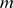 generations, before one's expenses would exceed the cost of becoming infected. When returns are diminishing, fewer than 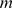 generations of total isolation are practical. Thus, the dimensionless efficiency 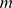 can be thought of as an upper bound on the number of transmission generations individuals can afford to isolate themselves before the costs of social distancing outweigh the costs of infection.

**Figure 2:**
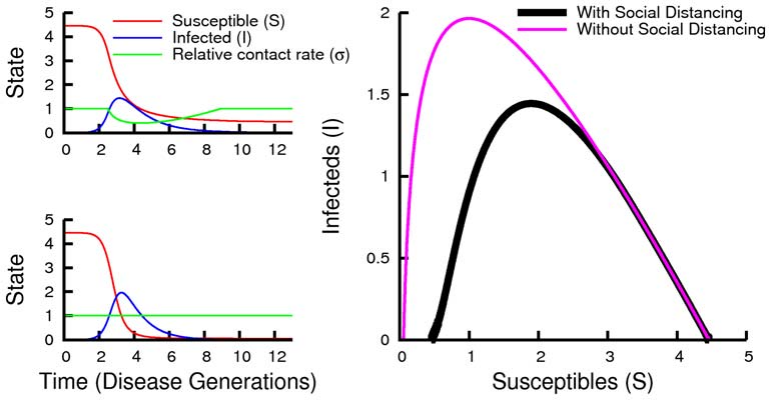
Epidemic solutions with equilibrium social distancing and without social distancing. Social distancing reduces the epidemic peak and prolongs the epidemic, as we can see by comparing a time series with subgame-perfect social distancing (top left) and a time series with the same initial condition but no social distancing (bottom left) (parameters 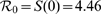, 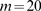). In the phase plane (right), we see that both epidemics track each other perfectly until 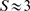, when individuals begin to use social distancing to reduce transmission. Eventually, social distancing leads to a smaller epidemic. The convexity change appearing at the bottom the phaseplane orbit with social distancing corresponds to the cessation of social distancing.

**Figure 3:**
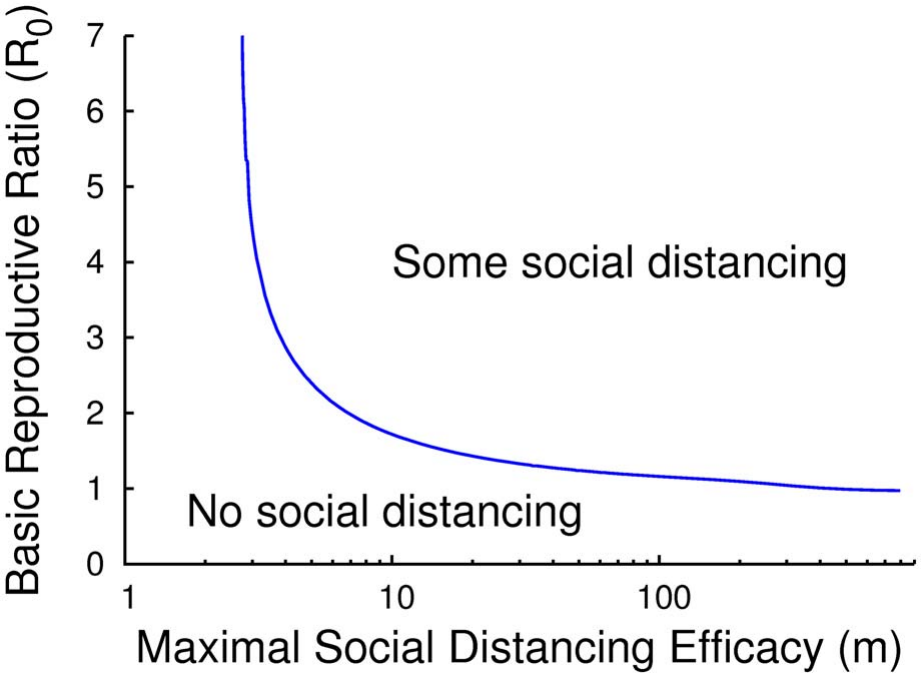
Social distancing threshold. This is the threshold that dictates whether or not equilibrium behavior involves some social distancing. It depends on both the basic reproduction number 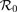 and the maximum efficiency 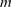, and is independent of the exact form of 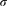. As rough rules of thumb, if 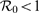 or 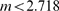, then equilibrium behavior involves no social distancing.

For the infinite-horizon problem, an example equilibrium strategy and the corresponding dynamics in the absence of social distancing are shown in Figure 2. We can show that if social distancing is highly inefficient (the maximum efficiency 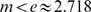), then social distancing is a waste of effort, no matter how large 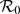. If social distancing is efficient, then there is a threshold value of 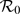 below which social distancing is still impractical because the expected costs per day to individuals is too small compared to the cost of social distancing, but above which some degree of social distancing is always part of the equilibrium strategic response to the epidemic (Figure 3).

The exact window over which social distancing is used depends on the basic reproduction number, the initial and terminal conditions, and the efficiency of distancing measures. The feedback form of equilibrium strategies, transformed from 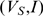 coordinates to the 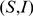 coordinates of the phase-space is represented with contour plots in Figure 1. Among equilibrium strategies, social distancing is never used until part-way into the epidemic, and ceases before the epidemic fully dies out.

**Figure 4:**
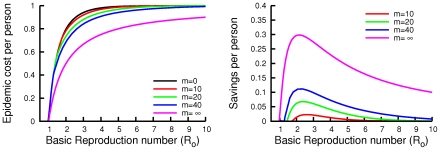
Total costs and savings. Plots of the total per-capita cost of an epidemic 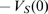 (left) under equilibrium social distancing for the infinite-horizon problem with several efficiencies 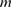 under Eq. (6), and the corresponding per-capita savings (right). Savings in expected cost compared to universal abstention from social distancing are largest for moderate basic reproduction numbers, but are relatively small, even in the limit of infinitely efficient social distancing. The 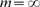 case corresponds to infection of the minimum number of people necessary to reduce the reproduction ratio below 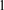.

The consequences of social distancing are shown in Figure 4. The per-capita cost of an epidemic is larger for larger basic reproduction numbers. The more efficient social distancing, the more of the epidemic cost can be saved per person. However, the net savings from social distancing reaches a maximum around 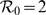, and never saves more than 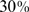
% of the cost of the epidemic per person. For larger 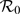
's, social distancing is less beneficial.

**Figure 5:**
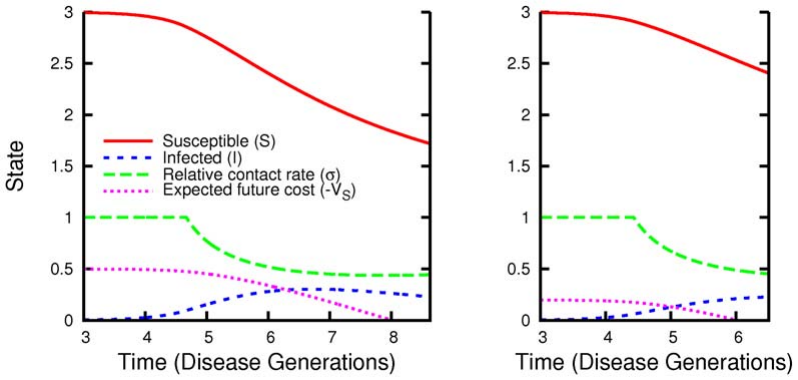
Solutions when vaccine becomes available after a fixed time. These are time series of an equilibrium solution for social distancing when mass vaccination occurs 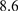 generations (left) and 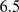 generations (right) after the start of the epidemic. Investments in social distancing begin well after the start of the epidemic but continue right up to the time of vaccination. Social distancing begins sooner when vaccine development is faster. For these parameter values (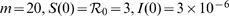), individuals save 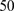
% of the cost of infection per capita (left) and 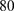
% of the cost of infection (right).

**Figure 6:**
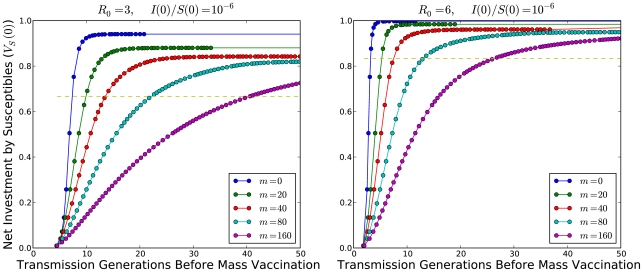
Windows of Opportunity for Vaccination. Plots of how the net expected losses per individual (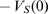) depend on the delay between the start of social-distancing practices and the date when mass-vaccination becomes universally available if individuals use a Nash equilibrium strategy. The more efficient social distancing, the less individuals invest prior to vaccine introduction. The blue lines (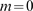) do not use social distancing, as the efficiency is below the threshold. The dotted lines represent the minimal asymptotic epidemic costs necessary to stop an epidemic.

We can also calculate solutions of the finite-time horizon problem where a vaccine becomes universally available at a fixed time after the detection of disease (Figure 5). If mass vaccination occurs soon enough, active social distancing occurs right up to the date of vaccination. Using numerical calculations of equilibria over finite-time horizons, we find that there is a limited window of opportunity during which mass vaccine can significantly reduce the cost of the epidemic, and that social distancing lengthens this window (Figure 6). The calculations show that increases in either the amount of time before vaccine availability or the basic reproduction number increase the costs of the epidemic. Smaller initial numbers of infections allow longer windows of opportunity. This is as expected because the larger the initial portion of the population infected, the shorter the time it takes the epidemic to run its full course.

## Discussion

Here, I have described the calculations necessary to identify the equilibrium solution of the differential game for social distancing behaviors during an epidemic. The benefits associated with the equilibrium solution can be interpreted as the best outcome of a simple social-distancing policy. We find that the benefits of social distancing are constrained by fundamental properties of epidemic dynamics and the efficiency with which distancing can be accomplished. The efficiency results are most easily summarized in terms of the maximum efficiency 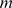, which is the percent reduction in contact rate per percent of infection cost invested per disease generation. As a rule-of-thumb, 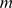 is an upper bound on the number of transmission generations individuals can isolate before the costs of social distancing outweigh the costs of infection. Social distancing is not practical if this efficiency is small compared to the number of generations in the fastest epidemics (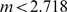). While social distancing can yield large reductions in transmission rate over short periods of time, optimal social-distancing strategies yield only moderate reductions in the cost of the epidemic.

Our calculations have determined the equilibrium strategies from the perspective of individuals. Alternatively, we could ask what the optimal social distancing practices are from the perspective of minimizing the total cost of the epidemic to the community. Determination of the optimal community strategy leads to a nonlinear optimal control problem that can be studied using standard procedures [23]. Yet, practical bounds on the performance of the optimal community strategy can be obtained without further calculation. The optimal community strategy will cost less than the game-theoretic solution per capita, but must cost more than 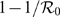, as that is the minimum number of people who must become sick to reduce the effective reproduction number below the epidemic threshold. Preliminary calculations indicate that optimal community strategies and game equilibrium strategies converge as 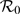 grows, and significant differences are only observable for a narrow window of basic reproduction numbers near 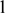.

The results presented require a number of caveats. I have, for instance, only considered one particular form for the relative risk function. Most of the analysis has been undertaken in the absence of discounting (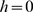), under the assumption that the epidemic will be fast compared to planning horizons. Discounting would diminish importance of long term risks compared to the instant costs of social distancing, and thus should diminish the benefits of social distancing. The benefits of social distancing will also be diminished by incorporation of positive terminal costs of vaccination (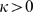). Realistically, mass vaccination cannot be accomplished all-at-once, as we assume. It's much more likely that vaccination will be rolled out continuously as it becomes available. This could be incorporated into our analysis, for instance, by including a time-dependent forcing. Other approaches include extending the model to incorporate vaccination results of Morton and Wickwire [28], or to allow an open market for vaccine purchase [18].

The simple epidemic model is particularly weak in its prediction of the growths of epidemics because it assumes the population is randomly mixed at all times. We know, however, that the contact patterns among individuals are highly structured, with regular temporal, spatial, and social correlations. One consequence of heterogeneous contact structure is that epidemics proceed more slowly than the simple epidemic model naively predicts. Thus, the simple epidemic model is often considered as a worst-case-scenario, when compared with more complex network models [29], [30] and agent-based models [31]–[33]. In the context of social distancing, it is not immediately clear how weaker mixing hypotheses will affect our results. Weakened mixing will prolong an epidemic, increasing the window over which social distancing is needed. But under weakened mixing, individuals may be able to use local information to refine their strategies in ways analogous to the ideas of Funk et al. [9] and Perisic and Bauch [34]. In general, the analysis of aggregate games with stochastic population dynamics require a significant technical leaps, and are the subjects of active research.

One of the fundamental assumptions in our analysis is that there are no cost-neutral behavior changes that can reduce contact rates. In fact, life-experience provides good evidence that many conventional aspects of human behavior are conditional on cultural norms, and that different cultures may adopt alternative conventions. The introduction of a new infectious disease may alter the motivational pressures so that behavioral norms that were previously equivalent are no longer, and that one norm is now preferred to the others. In such cases, there are likely to be switching costs that retard the rapid adoption of the better behaviors that conflict with cultural norms. The rate of behavior change, then, would be limited by the rate of adoption of compensatory changes in cultural norms that reduce the cost of social distancing.

Another deep issue is that behavior changes have externalities beyond influencing disease incidence, but we have not accounted for these externalities. People's daily activities contribute not just to their own well-being but also to the maintenance of our economy and infrastructure. Social distancing behaviors may have serious negative consequences for economic productivity, which might feed back into slowing the distribution of vaccines and increasing daily cost-of-living expenses.

We can extend our analysis to include economic feedbacks by incorporating capital dynamics explicitly. Individuals may accumulate capital resources like food, water, fuel, and prophylactic medicine prior to an epidemic, but these resources will gradually be depleted and might be difficult to replace if social distancing interferes with the economy flow of goods and services. Further capital costs at the community and state scales may augment epidemic valuations. These factors appear to have been instrumental in the recent US debate of school-closure policies. One feature of a model with explicit capital dynamics is the possibility of large economic shocks. This and related topics will be explored in future work.

These calculations raise two important mathematical conjectures which I have not attempted to address. The first is that the social distancing game possesses a unique subgame-perfect Nash equilibrium. There is reasonable numerical evidence of this in cases where the relative risk function 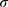 is strictly convex, and stronger unpublished arguments of this in cases of piecewise linear 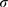. I believe this will also be the case for non-convex but monotone relative risks under some allowances of mixed-strategies. A second conjecture, not yet addressed formally, is that increases in the efficiency of social distancing always lead to greater use of social distancing, all other factors being equal. This seems like common sense, but the precise dependence of Figure 1 on the efficiency has yet to be determined mathematically.

As with all game-theoretic models, human behavior is unlikely to completely agree with our equilibria for many reasons, including incomplete information about the epidemic and vaccine and strong prior beliefs that impede rational responses. On the other hand, our approach is applicable to a large set of related models. We can analyze many more realistic representations of pathogen life-cycles. For instance, arbitrary infection-period distributions and infection rates can be approximated using a linear chain of states or delay-equations [24]. Structured populations with metapopulation-style mixing patterns may also be analyzed. I hope to apply the methods to a wider variety of community-environment interactions in the future.

## References

[1] Dawood F, Jain S, Finelli L, Shaw M, Lindstrom S (2009). Emergence of a novel swine-origin influenza A (H1N1) virus in humans.. New England Journal of Medicine.

[2] Del Valle S, Hethcote H, Hyman JM, Castillo-Chavez C (2005). Effects of behavioural changes in a smallpox attack model.. Mathematical Biosciences.

[3] Kelso J, Milne G, Kelly H (2009). Simulation suggests that rapid activation of social distancing can arrest epidemic development due to a novel strain of influenza.. BMC Public Health.

[4] Hyman JM, Li J (2007). Infection-age structured epidemic models with behavior change or treatment.. Journal of Biological Dynamics.

[5] Reluga TC, Medlock J (2007). Resistance mechanisms matter in SIRS models.. Mathematical Biosciences and Engineering.

[6] Reluga TC, Bauch CT, Galvani AP (2006). Evolving public perceptions and stability in vaccine uptake.. Mathematical Biosciences.

[7] Buonomo B, d'Onofrio A, Lacitignola D (2008). Global stability of an sir epidemic model with information dependent vaccination.. Mathematical Biosciences.

[8] Chen FH (2009). Modeling the effect of information quality on risk behavior change and the transmission of infectious diseases.. Mathematical Biosciences.

[9] Funk S, Gilad E, Watkins C, Jansen V (2009). The spread of awareness and its impact on epidemic outbreaks.. Proceedings of the National Academy of Sciences.

[10] Epstein J, Parker J, Cummings D, Hammond R (2008). Coupled contagion dynamics of fear and disease: mathematical and computational explorations.. PLoS ONE.

[11] Fine PEM, Clarkson JA (1986). Individual versus public priorities in the determination of optimal vaccination policies.. American Journal of Epidemiology.

[12] Bauch CT, Galvani AP, Earn DJD (2003). Group interest versus self-interest in smallpox vaccination polic y.. Proceedings of the National Academy of Sciences.

[13] Chen FH (2006). A susceptible-infected epidemic model with voluntary vaccinations.. Journal of Mathematical Biology.

[14] Reluga TC, Medlock J, Poolman E, Galvani AP (2007). Optimal timing of disease transmission in an age-structured population.. Bulletin of Mathematical Biology.

[15] Toxvaerd F (2009). The economics of infectious disease: Infection, treatment and acquired immunity..

[16] d'Onofrio A, Manfredi P (2009). Information-related changes in contact patterns may trigger oscillations in the endemic prevalence of infectious diseases.. Journal of Theoretical Biology.

[17] Reluga TC (2009). An SIS game with two subpopulations.. Journal of Biological Dynamics.

[18] Francis PJ (2004). Optimal tax/subsidy combinations for the flu season.. Journal of Economic Dynamics and Control.

[19] van Boven M, Klinkenberg D, Pen I, Weissing F, Heesterbeek H (2008). Self-Interest versus Group-Interest in Antiviral Control.. PLoS ONE.

[20] Isaacs R (1965). Differential Games: A mathematical theory with applications to warfare and pursuit, control and optimization.

[21] Arrow KJ, Kurz M (1970). Public Investment, the Rate of Return, and Optimal Fiscal Policy.

[22] Clark C (1976). Mathematical bioeconomics.

[23] Lenhart S, Workman J (2007). Optimal Control Applied to Biological Models.

[24] Reluga TC, Galvani AP (2010). A general approach for population games with application to vaccination..

[25] Kermack WO, McKendrick AG (1927). Contributions to the mathematical-theory of epidemics.. Proceedings of the Royal Society of London.

[26] Chen FH (2004). Rational behavioral response and the transmission of stds.. Theoretical Population Biology.

[27] Reluga TC, Medlock J, Galvani AP (2009). The discounted reproductive number for epidemiology.. Mathematical Biosciences and Engineering.

[28] Morton R, Wickwire KH (1974). On the optimal control of a deterministic epidemic.. Advances in Applied Probability.

[29] Meyers LA, Newman ME, Pourbohloul B (2006). Predicting epidemics on directed contact networks.. Journal of Theoretical Biology.

[30] Meyers LA (2007). Contact network epidemiology: Bond percolation applied to infectious disease prediction and control.. Bulletin of the American Mathematical Society.

[31] Mniszewski S, Del Valle S, Stroud P, Riese J, Sydoriak S (2008). EpiSimS simulation of a multi-component strategy for pandemic influenza.. Proceedings of the 2008 Spring simulation multiconference.

[32] Longini IM, Nizam A, Xu S, Ungchusak K, Hanshaoworakul W (2005). Containing pandemic influenza at the source.. Science.

[33] Ferguson N, Cummings D, Cauchemez S, Fraser C, Riley S (2005). Strategies for containing an emerging influenza pandemic in Southeast Asia.. Nature.

[34] Perisic A, Bauch C (2009). Social Contact Networks and Disease Eradicability under Voluntary Vaccination.. PLoS Computational Biology.

